# Population Characteristics in a Tertiary Pain Service Cohort Experiencing Chronic Non-Cancer Pain: Weight Status, Comorbidities, and Patient Goals

**DOI:** 10.3390/healthcare5020028

**Published:** 2017-06-14

**Authors:** Katherine Brain, Tracy Burrows, Megan E. Rollo, Chris Hayes, Fiona J. Hodson, Clare E. Collins

**Affiliations:** 1 School Health Science, Faculty of Health and Medicine, University of Newcastle, Callaghan, NSW 2305, Australia; katherine.brain@uon.edu.au (K.B.); tracy.burrows@newcastle.edu.au (T.B.); megan.rollo@newcastle.edu.au (M.E.R.); 2 Priority Research Centre in Physical Activity and Nutrition, University of Newcastle, Callaghan, NSW 2305, Australia; 3 Hunter Integrated Pain Service, Newcastle, NSW 2300, Australia; chris.hayes@hnehealth.nsw.gov.au (C.H.); fiona.hodson@health.nsw.gov.au (F.J.H.)

**Keywords:** weight, nutrition, pain, lifestyle

## Abstract

We describe the characteristics of patients attending an Australian tertiary multidisciplinary pain service and identify areas for nutrition interventions. This cross-sectional study targets patients experiencing chronic pain who attended the service between June–December 2014. Self-reported data was captured from: (1) an Electronic Persistent Pain Outcomes Collaboration (ePPOC) referral questionnaire, incorporating demographics, pain status, and mental health; (2) a Pain Assessment and Recovery Plan (PARP), which documents patients’ perceived problems associated with pain and personal treatment goals. The ePPOC referral questionnaire was completed by 166 patients and the PARP by 153. The mean (SD) patient age was 53 ± 13 years, with almost 60% experiencing pain for >5 years. Forty-five percent of patients were classified as obese (BMI ≥ 30 kg/m^2^, mean (SD) BMI was 31 ± 7 kg/m^2^), with a mean waist circumference of 104 ± 19.4 cm (SD). The most frequent patient nominated treatment goals related to physical activity (39%), followed by nutritional goals (23%). Traditionally, pain management programs have included physical, psychosocial, and medical, but not nutritional, interventions. By contrast, patients identified and reported important nutrition-related treatment goals. There is a need to test nutrition treatment pathways, including an evaluation of dietary intake and nutrition support. This will help to optimize dietary behaviors and establish nutrition as an important component of multidisciplinary chronic pain management.

## 1. Introduction

Chronic pain is defined as pain that persists beyond the usual time for tissue healing, or pain that continues beyond three to six months [1]. Musculoskeletal conditions and neurological injuries are commonly associated with chronic pain [2]. In Australia, osteoarthritis is the most common structural condition associated with chronic pain [2]. However approximately 30% of those who experience chronic pain have no obvious structural contributors and a large body of pain research has sought an explanation for this [3]. Neuroscience research has helped to provide answers with important insights into the contribution of nervous system sensitization and brain interpretation in the expression of chronic pain [4].

Approximately 3.2 million Australians experience chronic pain and this is estimated to rise to 5 million by 2050 as the population ages [3]. Chronic pain often occurs with other physical and mental health comorbidities including depression and anxiety, heart disease, and diabetes [5]. Current treatment services in Australia include over 50 public and private multidisciplinary pain management services, which typically provide a range of interventions including group-based pain management programs [6]. Treatments commonly include reducing the reliance on medication (such as opioid de-prescribing) and lifestyle-based interventions, including cognitive approaches and physical activity. In contrast, nutritional expertise is commonly lacking within multidisciplinary teams. Of the 20 services located in NSW, Australia, three list nutrition as part of the program content provided to patients, but none have a nutrition expert employed as part of their team [7].

Research has only partially explored the relationship between nutritional status and chronic pain. Some evidence suggests an association between chronic pain and poor diet quality with higher intakes of energy-dense, nutrient poor foods [8,9]. There are a number of studies that explore the association between obesity and chronic pain, including a recent systematic review summarizing the evidence which supports the notion that there is a higher prevalence of chronic pain in people who are obese, compared to a normal weight population [10]. This review further emphasizes the importance of including weight reduction in chronic pain management [10]. Other studies have also reported a link between greater pain perception and a higher weight status, with individuals classified as obese twice as likely to experience pain [11,12]. Stone et al. present the results from a large US-based population study (*n* > 1,000,000) and when controlling for age, gender, race, education, smoking, and the presence of health care coverage, the associated risk for experiencing pain was 3.5 times greater for those who were in the obese III (BMI ≥ 40 kg/m^2^) category compared to those in the normal BMI (18.5–24.99 kg/m^2^) category [12]. It has been suggested that the relationship between a poor nutrition status and chronic pain may in part be mediated by nervous and immune system sensitization [13,14]. In addition, excess body weight can contribute to pain through a direct mechanical load on specific joints [15]. Both Okifuji et al. and Ding et al. outline a number of issues, including: the association between a higher BMI and pain and a significant association between a higher BMI and the prevalence of defective knee cartilage [15,16].

The current study aims to summarize and describe the demographics, pain characteristics, weight status, comorbidities, and treatment goals of patients attending an Australian multidisciplinary chronic pain service. This study will identify the prevalence of overweight and obesity and explore patient treatment goals in a real-world clinical population. This will enable the identification of major nutrition-related issues, as reported by patients that could be used to inform appropriate treatment and the future development of tailored interventions.

## 2. Materials and Methods 

**Figure 1:**
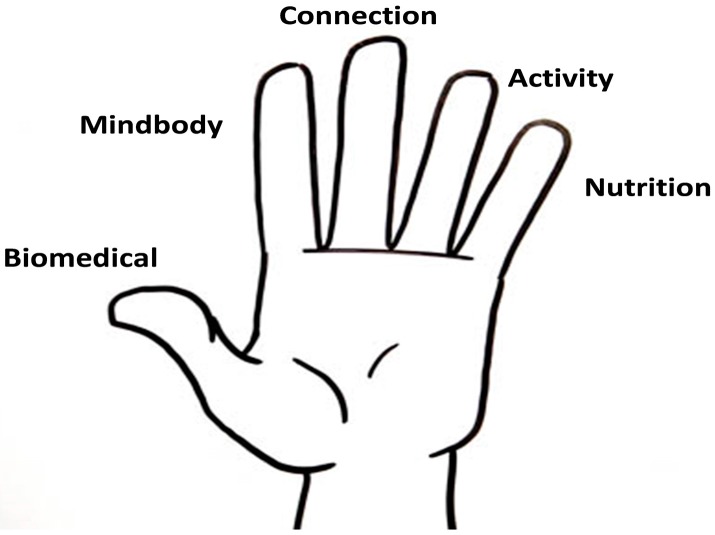
Whole person approach to pain management, Hunter Integrated Pain Service.

This descriptive cross sectional study was undertaken at Hunter Integrated Pain Service (HIPS), which provides a person-centered approach to pain management incorporating aspects of biomedicine, mindbody, connection, physical activity, and basic nutrition education (Figure 1) [17]. In practical terms for patients experiencing chronic non-cancer pain, this involves a shift from the passive receipt of medical treatment toward active lifestyle changes. Opioid de-prescribing is an important part of this approach. The treatment programs offered at HIPS are currently facilitated by pain medicine specialists, nursing staff, psychologists, and physiotherapists. However there are no dietetic staff within the service. Referrals to the service for chronic non-cancer pain are generally made by the patient’s general practitioner or medical specialist. Patients are then invited to attend a seminar, Understanding Pain (UP), which outlines current pain science and an overview of the service. Patients then have a choice to either leave the service (where they continue with their GP and community services) or be triaged into one of two pathways. The first pathway is the group-based pathway and this is the path that the majority of patients follow; it begins with a group-based assessment and planning workshop (A&P), followed by a six week group-based treatment program called Active Pain Treatment (APT). Patients then have the choice to attend a follow up session called Progress Review Group and/or a Mindfulness Group. The second pathway involves individual management. Patients may be triaged to attend an individualized multidisciplinary appointment or one-on-one appointment with a specific clinician. Other patients may attend a procedural appointment. Those who attend an individualized or procedural appointment are strongly encouraged to follow up by attending the group-based treatment options.

Patients who attended HIPS between June–December 2014 were identified by searching the Electronic Persistent Pain Outcomes Collaboration (ePPOC) database [5]. The Medical Record Numbers (MRN’s) were extracted and used to search Digital Medical Records (DMR) to find patients eligible for inclusion in the study. To be eligible for inclusion, patients had to have: (1) a completed ePPOC referral questionnaire, which patients complete before attending UP; (2) a Pain Assessment and Recovery Plan (PARP), completed at A&P, on file; and (3) have provided consent for their data to be used in research projects via the ePPOC referral questionnaire, which included a consent statement. During data extraction, it was identified that a portion of these patients had completed an earlier version of the PARP which could not be merged due to the qualitative nature of the data. These PARPs were excluded from the analysis.

The ePPOC referral questionnaire consists of eight sections and was completed by patients before they entered the pain service. Section 1 covers demographic questions (21 items), including: gender, date of birth, country of birth, ethnicity, and work status (where possible answers (*n* = 11) ranged from retired to full time work). In addition, Section 1 asked general questions relating to pain status such as the cause of pain (where participants could nominate their perception of the cause of their pain, choosing from eight pre-defined categories ranging from injury to cancer) and the main pain site on the body. The ePPOC survey also asked for details on height, weight, and comorbidities (where participants could choose from one or more responses (*n* = 13) and the possible answers ranged from kidney disease to osteoarthritis). The ePPOC survey also included a question asking if patients required assistance filling out the form with the option of a yes/no answer. Section 2 and Section 3 relate to the use of health services (six items) and current medication (one item). Sections 4–7 include standardized, validated pain assessment tools including the Brief Pain Inventory (BPI) [18,19], Pain Self Efficacy Questionnaire (PSEQ) [20], Pain Catastrophising Scale (PCS) [21], and the Depression Anxiety Stress Scale 21 (DASS-21) [22]. The BPI, PSEQ, and PCS describe pain severity and interference, confidence in carrying out activities despite pain, and thoughts and feelings associated with pain, respectively. The BPI severity and interference score is rated on a scale of 0–10 for severity, where a score of 0–4 indicates mild pain, 5–6 moderate pain, and 7–10 severe pain. An average of the seven interference questions is calculated as a score out of 10, with the higher the score, the higher the interference. The PSEQ total is a sum of the scores (0 = not confident at all to 6 = completely confident) from 10 questions. A higher score indicates a higher level of self-efficacy: severe < 20, moderate 20–30, mild 31–40, and minimal impairment > 40. The PCS measures pain catastrophising by measuring three sub-categories: rumination, magnification, and helplessness. A score of <20 indicates mild catastrophising, high is 20–30, and severe is >30. The DASS-21 provides a score for each domain of depression (normal 0–9, mild 10–13, moderate 14–20, severe 21–27, extremely severe 28+), anxiety (normal 0–7, mild 8–9, moderate 10–14, severe 15–19, extremely severe 20+), and stress (normal 0–14, mild 15–18, moderate 19–25, severe 26–33, extremely severe 34+), and classifies each patient from normal to extremely severe. Where possible, survey data (BPI, PSEQ, and DASS-21) was scored according to pre-specified author instructions. Section 8 (10 items) includes additional information such as other health professionals involved, previous medication use, smoking, and alcohol and caffeine consumption.

As a patient centered treatment plan that facilitates goal setting, the PARP questionnaire was developed by the HIPS team to encourage the use of active self-management skills to treat pain. This tool allows patients to select perceived problems associated with their pain experiences, set individualized goals, and nominate the solutions that they would like to pursue. Problems and solutions can be selected from five areas: biomedical, mindbody, connection, physical activity, and nutrition. The biomedical domain considers the balance of body structure and nervous system contributions to pain along with medication use, mindbody addresses thoughts and emotions related to pain, and connection explores the linkage with people, places, and purpose. The activity domain covers the patient’s ability to undertake physical activity and reduce sedentary behaviour. The nutrition section provides an opportunity for patients to self-report waist circumference and patients are provided with a tape measure and brief instructions on how to do this. Patients can also list any intention to focus on a balanced diet and/or other strategies (e.g., reduce sugar intake and increase water and/or fruit and vegetable consumption) to improve diet quality.

Ethics approval for the current study was obtained from Hunter New England Health (HNEH) (15/07/15/5.01) and the University of Newcastle (H-2015-0266).

### Data Analysis

Data were extracted from each survey and linked via patient MRNs. The date of birth was obtained to calculate the age of the participants and this was subsequently collapsed into 20 year age brackets. Height and weight were used to calculate BMI using standardized equations. Where patients were able to list “other” comorbidities, the answers were collated and those which fell under one of the pre-existing comorbidities were moved to that group. Patient goals were categorized and collated based on the five domains in the PARP. A patient’s BPI severity, BPI interference, PSEQ, PCS, and nutrition-related goals were collated for those patients with adequate data to allow a BMI calculation. This data was then compared based on the BMI category (normal weight, overweight, and obese). Sample statistics were used to explore associations between these variables. BPI severity and PCS were analysed using ANOVA, while BPI interference and PSEQ were analysed using a non-parametric Kruskal Wallis test. A chi-squared test was used to compare BMI categories and the % patients who selected a nutrition-related goal. For those results which were statistically significant (*p* < 0.05), post-hoc testing was carried out. All statistics were generated using Stata13 [23] and descriptive statistics were reported as mean ± standard deviation or response frequencies and sample statistics reported using *p*-values.

## 3. Results

A total of 166 patients consented for their data to be used in research and had a complete ePPOC referral questionnaire at the time of entry to the service, which was subsequently included in the study. This is just over one third of the total patient cohort that HIPS would see, at any stage of treatment, in a six month period. Of these, 93% (*n* = 153) of patients also completed the appropriate PARP, which was analysed separately. Thirteen patients had insufficient data due to the completion of an earlier version of PARP that could not be merged.

### 3.1. Patient Demographics

Information provided via the ePPOC referral questionnaire identified that 57% of patients were female (Table 1), with a mean age of 53 ± 13 years (SD) (range 21–89 years) and no differences in the demographic characteristics by gender. The major age group was 41–60 years, incorporating 55% of patients. Ninety percent of patients were born in Australia, with 5% identifying as being Aboriginal or Torres Strait Islander (*n* = 8) and 1% being of Maori descent (*n* = 1). Thirty seven percent described their work status as *unemployed (due to pain)* and listed *osteoarthritis/degenerative arthritis* (25%) and *depression/anxiety* (22%) as the top two most common comorbidities experienced in addition to pain. Twelve percent chose the comorbidity category “*other, please specify*”, with 40% specifying a pain-related condition, and of this, 28% listed fibromyalgia. Other comorbid conditions included asthma (8%) and sleeping difficulties (8%). Thirty six percent of patients reported having <2 comorbidities (from the 13 listed categories), and 64% of patients reported ≥2 comorbidities [24]. On average, each patient reported taking eight medications (range 0–31), with a total of 1356 medications listed by the 166 patients. Seventy-one percent of patients reported ≥1 opioid, 69% paracetamol, 51% antidepressant, 42% anticonvulsant, 31% non-steroidal anti-inflammatory (NSAID), and 28% a nutrition related supplement. Approximately one quarter of the patients were taking ≥1 medication for hypertension and hypercholesterolemia and 10% were taking ≥1 laxative, with one patient reporting four laxatives. Of the 1356 medications listed, 33% related to pain relief, 9% were a nutritional supplement, 9% were antidepressants, 7% were anticonvulsants (which may or may not be directed toward the treatment of neuropathic pain), 6% were for treating hypertension, 3% were for treating high cholesterol, and 2% were laxatives.

Based on the data provided by the DASS-21 tool, 81% of patients had some degree of depression (9% mild, 20% moderate, 16% severe, and 36% extremely severe). The anxiety component of the DASS-21 showed that 57% of patients had some degree of anxiety (7% mild, 14% moderate, 12% severe, and 24% extremely severe). Similarly, the stress component showed that 76% of patients had some level of stress (9% mild, 20% moderate, 24% severe, and 23% extremely severe). The average (SD) score for each component was 21.75 ± 12.57, 14.62 ± 10.60, and 20.67 ± 11.44, respectively.

### 3.2. Patients’ Description of Pain Experience

A total of 185 answers to the question “*what was the cause of your pain?*” were selected by the 166 patients. The top answer for patient perceived causes of pain was *injury at work/school* (24%) and the main pain site selected by patients was the back (40%) (Table 2). Just under half of the patients (48%) stated that they had pain in one to three body sites. Eighty four percent of patients described their pain *as always present, but at varying intensity*. The majority (58%) of patients stated that they had experienced pain for more than five years. The majority of patients (71%) reporting taking ≥1 opioid-based medication. Of the total medications listed by patients, 33% were related to pain relief: 16% were opioids, 6% paracetamol, 5% NSAIDS, and 5% combination analgesic (paracetamol/NSAID and codeine). The BPI was completed by 161 patients with the pain severity score: 21% mild, 42% moderate, and 35% severe. The mean BPI severity score was 6.32 ± 1.72 (range 1.3–10) and the BPI interference score was 7.32 ± 2 (range 0.3–10) out of a possible 10. The PSEQ categorizes pain self-efficacy and the average (SD) score was 19.59 ± 12.01, with 6% rated as having minimal impairment, 13% mild, 26% moderate, and 55% severe. The average PCS score was 30.07 ± 13.10, which just falls into the severe category. Just over half (51%) of patients fell into the severe category, with 25% falling into the high category and 24% into the mild category.

In terms of health service use over the preceding three months, patients had visited their GP a mean of 4.5 times and saw a health professional (other than a doctor) three times due to pain. There were 280 professionals listed by 107 patients in response to the question “*What health professionals are you seeing?*”. These professionals can be categorized into 43 professions. The top professionals (excluding “other”) that were listed included general practitioners, physiotherapists, psychologists, and surgeons (12.5%, 11%, 10%, and 7% respectively). The least commonly reported was a dietitian, which was listed by one patient.

### 3.3. Patients’ Nutrition-Related Health and Treatment Goals

**Figure 2:**
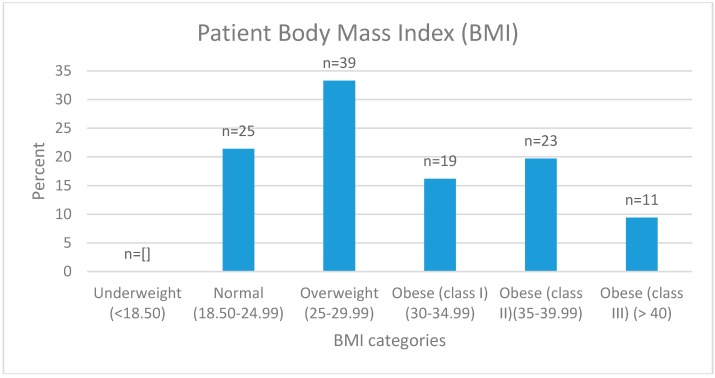
Body Mass Index (BMI) of patients attending the Hunter Integrated Pain Service.

A total of 117 patients had anthropometric data recorded. The mean BMI was 31 ± 7 kg/m^2^ (range 18.52–54.46 kg/m^2^) (Figure 2). According to WHO classifications, 21% of patients were in the normal BMI category (18.5–24.99 kg/m^2^), 33% were in the overweight category (25–29.99 kg/m^2^), and 45% were in the obese category (≥30 kg/m^2^). The average BMI of those taking opioids (30.96 kg/m^2^) and not taking opioids (29.71 kg/m^2^) was similar. The mean waist circumference (reported by *n* = 138) was 104 ± 19.40 cm (range 66–165 cm). Of these, 82 females reported a waist circumference with a mean of 101.21 ± 19.70 cm (range 66–150 cm) and males (*n* = 56) 108.61 ± 18.23 cm (range 82.5–165 cm). Eighty seven percent of females and 77% of males recorded waist circumferences that categorized them (≥80 cm and ≥94 cm respectively) as at risk of developing chronic disease [25]. Of the 1356 medications listed by the patients, 9% were either a vitamin, mineral, omega-3, or combination of the three.

Most patients reported that they drank alcohol less than one day per week (70%), with 3% stating that they drank every day of the week. Of those who did drink alcohol, 57% indicated that they consumed one to two drinks per day and 6% reported having eight to 15 drinks per day. Fourteen percent of patients reported that they used alcohol to relieve pain, 42% said that they did not, and 44% did not answer this question. The majority of patients (54%) reported that they consumed one to three caffeinated drinks (coffee, cola, energy drinks) per day, with 2% stating that they had >8 per day.

Based on the data provided from the PARP, 34–39% of patients chose to make a nutritional change. These changes included: reducing the sugar intake, and increasing the intake of water, fruit, and vegetables. In addition, 13% of patients selected “referral to a dietitian” as a service needed to assist them with changes to their nutrition-related health. 

**Figure 3:**
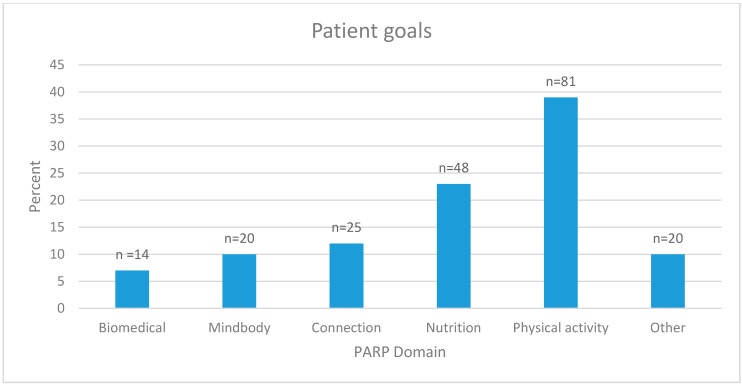
Patient goals, as defined by the five domains of treatment, Hunter Integrated Pain Service.

Patients were able to list and prioritize a treatment goal which could be selected from the five PARP domains (i.e., biomedical, mindbody, connection, physical activity, and nutrition). Of the 153 patients who completed the PARP, 141 set one or more goal(s). In a descending order of frequency, patients chose the following: physical activity (e.g., increase walking and strengthening exercise), nutrition (e.g., improve diet and lose weight), connection (e.g., to family, work and community), mindbody (e.g., seek psychological help), and biomedical (e.g., reduce opioid use) (Figure 3). Ten percent of goals could not be categorized into a domain (e.g., “improve what I am already doing” or “become pain free”). From those who listed nutrition, 27% stated that they wanted to improve their overall diet/nutrition, 47% chose a specific nutrition-related goal (e.g., reduce soft drink or sugar consumption, reduce portion size, and increase vegetable or water intake), and 27% stated that they wanted to reduce their weight or waist circumference.

Patients who had a normal weight BMI (18.5–24.99 kg/m^2^), overweight BMI (25–29.99 kg/m^2^), and obese (>30 kg/m^2^) were compared in terms of their pain-related scores (BPI severity, BPI interference, PSEQ, and PCS) and nutrition-related goals (Table 3). There were no statistically significant differences in BPI severity (*p* = 0.79), PCS (*p* = 0.93), or number of nutrition-related goals (*p* = 0.84) selected by patients by weight category. Statistically significant differences were found for BPI interference (*p* = 0.02) and PSEQ (*p* = 0.04) by weight status group. However, a post-hoc analysis indicated that the only significant difference in BPI interference was between those in the overweight versus obese groups (*p* < 0.001).

## 4. Discussion

This study has summarized the demographic data, pain characteristics, and nutritional status of patients attending HIPS. It was identified that almost 60% patients were female and the most common age group was middle aged adults aged 41–60 years. Almost 40% of patients stated that they were unemployed due to the impact of their pain experiences. This data reflects the same patient characteristics from a national database collected using the ePPOC tool in 21 pain services across Australia and presented in a 2014 report, where 57% of patients were female, with an average age of 53 years, and 36% reported that they were unemployed due to pain [26]. In a population of healthy young adults, unemployment was associated with a poor quality of life and inequality in terms of health status [27]. Considering the high level of unemployment in the current population, as well as complex health issues, it is highly likely that this cohort also have a poorer quality of life.

Just under a quarter (22%) of patients in this study reported depression and anxiety as a comorbid condition. When compared to data provided by the DASS-21, there is a large difference, with 81% and 57% of patients experiencing a degree of depression and anxiety, respectively. The discrepancy between these two results may be explained by the way in which the data was collected. Patient comorbidity is a self-reported measure which relies on the patient’s awareness and honesty, whereas the DASS-21 is a validated objective tool which is more accurate in identifying levels of depression and anxiety. In addition, those who were categorized with depression or anxiety via the DASS-21 may not have been formally diagnosed and therefore did not list these conditions as a comorbidity. When compared to national data, a similar trend exists, where over one third (34%) reported depression and anxiety as a comorbid condition [26] while 76% recorded a degree of depression and 67% anxiety on DASS-21 findings [26]. A literature review exploring the coexistence of chronic pain and depression found 15 studies where data was analysed from pain clinics and inpatient pain programs (number of patients ranged from 37–900) [28]. The percentage of patients with depression ranged from 1.5% to 100%, with six out of 15 studies having >40% of their patients reporting depression [28]. The high numbers of people who experience both chronic pain and depression contributes to poorer treatment responses and higher health care costs [29].

The most common pain site listed by patients attending HIPS reflects national data, with 43% in both populations selecting back pain [5]. Nationally, the second most common site was the shoulder region, whereas at HIPS, it was reported by patients as being leg pain. There was a greater proportion of HIPS patients who had experienced pain for more than five years (58%) compared to 48% of national patients [5]. Slightly more HIPS patients (55%) found that pain interfered with their self-efficacy compared to national data (52%). BPI scores were similar across both groups, with most patients describing pain intensity and interference as moderate or severe, respectively [26].

A large percentage (71%) of patients at HIPS reported taking ≥1 opioid medication, compared to the national cohort in 2014, where 61% patients were taking opioids on ≥2 days/week. The frequency of opioid consumption in the HIPS population is unable to be determined in this study, which is a limitation. There was also no difference between the BMI in those taking opioids and those not taking opioids, which suggests that there is no linear relationship between weight status and opioid consumption. It also suggests that there may not be a difference in the dietary status based on opioid consumption. The results found in Meleger et al. may apply to patients who experience chronic pain and do not take opioids [8]. Interestingly, after pain-related medication, nutrition supplements were the next highest group of medications to be consumed by patients. This highlights that further investigation is required to identify the potential benefits that nutrition and nutritional supplements can play for people who experience pain.

The average BMI and percentage of patients who fell into the overweight or obese category was higher at HIPS compared to the national pain service data [26] and the general population in Hunter New England Health Local Health District (HNELHD) [30]. However the data is based on self-report measures, so bias may exist. The average BMI of patients in this study was 31 ± 7 kg/m^2^ (obese category) compared to 28.9 ± 7.4 kg/m^2^ (overweight category) for the national pain services cohort in Australia. The study showed that 78% of the patients were overweight or obese, which is 115% higher than the national pain service data and 124% higher than HNELHD data. A wider gap exists between these populations when looking at obesity alone. Forty five percent of patients were obese, which is 122% higher than the national pain service data and 167% higher than HNELHD data. This study found that 21% of patients were in the normal (18.5–24.99 kg/m^2^), 33% in the overweight (25–29.99 kg/m^2^), and 45% in the obese (≥30 kg/m^2^) BMI category. In comparison, the national ePPOC report states that 3% patients fell into the underweight BMI category (<18.5 kg/m^2^), 29% in the normal, 31% in the overweight, and 37% in the obese category [26]. In 2014, in the Hunter New England Health Local Health District, 36% of the general population was overweight and 27% was obese [30]. When comparing these three populations, the difference in the percentage of people who are overweight is narrow, whereas the difference when focusing on those who are obese is significant. When comparing pain-related outcomes by BMI category, those in the obese category reported higher pain interference compared to those who were overweight (*p* < 0.001). All other results were not significant, suggesting that in this cohort, body weight is not significantly associated with the severity of most pain-related outcomes. The results from the current audit of a clinical population are not consistent with the current literature, where studies have shown a direct relationship between an increasing weight status and poorer pain experience [12,31,32]. A limitation of the current study is that it was not powered to detect statistically significant differences in outcomes by weight status, and hence, further research to explore the relationship between weight status and the characteristics of pain experiences in greater depth is warranted. Interestingly, there was no difference by the weight status group in the percentage of patients who chose a nutrition-related goal based on weight status. This suggests that patients consider nutrition an important part of their pain experience and treatment, irrespective of weight. Further research is needed to explore this outcome.

There is a substantial difference in the waist circumference between patients at HIPS and the general population of HNELHD. The average waist circumference for females attending HIPS compared to HNELHD was 101.2 cm and 87.5 cm, respectively. For males, the mean waist circumference was 108.6 cm at HIPS and 97.5 cm at HNELHD. The percentage of females considered “at risk” of developing chronic disease based on their waist circumference from the HIPS cohort and the HNELHD is 87% and 65.4% respectively. For males, 77% of the HIPS cohort and 59% of the HNELHD cohort had a waist circumference over the guidelines. In both females and males, the HIPS cohort had a higher percentage “at risk”, compared to the HNELHD.

The relationship between obesity and chronic pain is complex, with a higher weight status being a risk factor for developing chronic pain [15]. Conversely, overweight and obesity can be a result of pain, in association with limited mobility and poor eating habits [15]. This relationship needs to be investigated further in order to develop effective strategies to address the concurrence of these conditions. Patients at HIPS reported multiple and complex comorbidities, with almost two-thirds of patients reporting two or more comorbidities. There are many inter-relationships between comorbidities and chronic pain, which increases the complexity of the experience and the challenges faced when treating it. Most commonly, chronic pain is linked to mental health disorders and sleep disorders [33,34]. Mood and poor sleep play a huge role in a person’s experience of chronic pain [33,34], and in this study, both depression and sleep disorders have been reported by patients. There is also a high prevalence of the co-occurrence of chronic pain, depression, and cardiovascular disease [35]. Approximately one-fifth of the patients in this study reported having high blood pressure, diabetes, and heart disease, all of which contribute to cardiovascular disease and all of which are mediated by diet. Chronic pain can be considered a disease in its own right, with changes in the nervous system often becoming more important contributors than the original pain-related condition or injury [36]. As such, the initial pain-related condition or injury could be considered a comorbid condition. While some patients may be unaware of this differentiation, a proportion (14%) reported a specific pain-related condition when asked about their other medical conditions. This further emphasizes the complexity of chronic pain and the need for tailored education. HIPS patients report poorer health and are more likely to be socially disadvantaged compared to national data, which may explain why they have higher rates of overweight and obesity.

The study results indicate that dietary strategies that address personal nutrition-related problems were commonly chosen by patients, along with other lifestyle-related goals. Lifestyle-related goals comprised 62% of the goals chosen by patients, with nutrition selected as a target for one quarter of these patients. In contrast, only one patient had ever been referred to a dietitian. This highlights that there is a major disparity between the expressed needs of the patients and resources currently available within the health system to support those needs. This could be due to lengthy waiting lists for public dietetic services, a lack of awareness of alternative dietetic services in the community, and/or perceived expense for private health services. This disparity is also present in current literature as there is limited, yet growing support to include nutrition in chronic pain management services, particularly to support and complement physical activity in weight management [10,37]. However, this has yet to be followed up with feasibility and efficacy studies. Regardless of the reason, these disparities support prioritizing the integration of nutrition-related intervention into multidisciplinary pain services.

There are several limitations of the current study. Firstly, this was a one-off measure which limits the validity of drawing causal relationships. Secondly, the use of self-reported measures may be a source of bias. Height and weight data are self-reported by the patients as they fill in the questionnaire. However, self-report has been previously shown to be valid in other studies, including in a population of young adults [38]. Patients also measured their own waist circumference and therefore the results should be interpreted accordingly. In addition, this study was conducted at a single site with a relatively small number of patients. Hence the results may not be representative of other pain services in Australia. The strengths of this study include the use of clinical data routinely collected within a multidisciplinary pain service, which includes a questionnaire with validated tools for pain and mental health.

Behaviour change can be challenging to achieve, especially in a population with chronic pain whom have complex health issues and diverse social backgrounds. In practice, multi-modal behavioral strategies (e.g., using a biopsychosocial approach) are used to maximize the likelihood of achieving treatment benefit. It is also important to consider that personalized interventions are important for populations such as those with chronic pain. The current study highlights the need for testing a comprehensive nutrition-based intervention as part of the overall treatment package for chronic pain and its comorbidities. Future research should evaluate the feasibility and effectiveness of such an approach.

## 5. Conclusions

The current study has identified that patients attending a chronic pain management service report nutrition as an area of need that is currently not met within the treatment of chronic non-cancer pain. Patients referred to HIPS were more likely to be overweight or obese compared to community norms or patients referred to other pain services across Australia. In addition many patients expressed a desire to make nutrition-related lifestyle changes. Within a self-management approach, patients are able to initiate such changes themselves. However, the dietetic staff required to address this in a comprehensive way and support the nutritional change process are currently lacking. The addition of dietetic expertise to the routine workforce of a multidisciplinary pain team could support patient self-management in the area of nutrition and enable the development of pain specific, appropriate resources and outcome measures.

## Figures and Tables

**Table 1:** Patient Demographics.

Patient Demographics					
Characteristic	N	%	Characteristic	N	%
***Gender***			***Comorbidities*** **^1^**		
Male	71	43	Osteoarthritis/degenerative arthritis	116	25.2
Female	95	57	Depression and anxiety	102	22.1
***Work status*** **^1^**			Other	56	12.2
Unemployed (due to pain)	76	36.5	High blood pressure	54	11.7
Retired	44	21.2	Stomach/ulcer	27	5.9
Home duties	30	14.4	Diabetes	22	4.8
Paid work (part time)	10	4.8	Blood disease	15	3.3
Unemployed (not due to pain)	9	4.3	Heart disease	17	3.7
Studying (school/university)	9	4.3	Lung disease	14	3.0
On leave from work (due to pain)	9	4.3	Rheumatoid arthritis	14	3.0
Paid work (full time)	8	3.9	Neurological condition	13	2.8
Voluntary work	7	3.4	Cancer	8	1.7
Working (limited hours/duties)	4	1.9	Kidney disease	3	0.7
Retraining	2	1.0			

^1^ Patients could select more than one answer.

**Table 2:** Patients’ description of pain experience.

Patients’ Description of Pain Experience					
Characteristic	N	%	Characteristic	N	%
***Cause of pain*^1^**			***Main pain site*^1^**		
Injury (work/school)	44	23.8	Back	65	40.1
Motor vehicle accident	24	13.0	Legs	28	17.3
No obvious cause	23	12.4	Neck	19	11.7
Other (not specified)	21	11.4	Arms/shoulder	12	7.4
Injury (other setting)	20	10.9	Head	8	4.9
Other illness	19	10.3	Feet	7	4.3
Surgery	19	10.3	Abdomen	5	3.1
Injury (home)	11	6.0	Knee	5	3.1
Cancer	4	2.2	Pelvis	4	2.5
***Frequency of pain*^1^**			Buttocks	3	1.9
Always present (varying intensity)	135	84.4	Hands	3	1.9
Always present (same intensity)	14	8.8	Chest	2	1.2
Often present	5	3.1	Whole body	1	0.6
Occasionally present	3	1.9	***Number of pain sites*^1^**		
Rarely present	3	1.9	1–3	76	47.5
***Time experiencing pain*^1^**			4–6	59	36.9
<3 months	3	1.9	7–9	21	13.1
3–12 months	11	6.9	>10	4	2.5
1–2 years	18	11.3			
2–5 years	34	21.4			
>5 years	53	58.5			

^1^ Patients could select more than one answer.

**Table 3:** Patients’ pain-related scores and nutrition-related goals, based on the BMI category.

	Normal BMI (*n* = 23)	Overweight BMI (*n* = 39)	Obese BMI (*n* = 51)
BPI (severity) (mean ± SD)	6.2 ± 2.0	6.0 ± 1.7	6.3 ± 1.7
BPI (interference) (mean ± SD)	7.3 ± 1.8	6.3 ± 2.3	7.6 ± 1.8
PSEQ (mean ± SD)	23.3 ± 10.9	22.5 ± 12.2	17.2 ± 12.0
PCS (mean ± SD)	28.0 ± 14.6	29.3 ± 12.8	28.8 ± 13.8
Nutrition-related goals (%)	30.4	25.6	31.4
